# Paediatric neck masses at a University teaching hospital in northwestern Tanzania: a prospective analysis of 148 cases

**DOI:** 10.1186/1756-0500-7-772

**Published:** 2014-11-03

**Authors:** Elibariki M Lucumay, Japhet M Gilyoma, Peter F Rambau, Phillipo L Chalya

**Affiliations:** Department of Surgery, Catholic University of Health and Allied Sciences-Bugando, Mwanza, Tanzania; Otorhinolaryngology & Head/Neck Surgery, Bugando Medical Centre, Mwanza, Tanzania; Department of Pathology, Catholic University of Health and Allied Sciences-Bugando, Mwanza, Tanzania

**Keywords:** Pediatrics, Neck masses, Patterns, Predictors, Outcome, Tanzania

## Abstract

**Background:**

Pediatric neck masses are one of the common surgical conditions presenting to the pediatric surgical wards and clinics in many centers worldwide. There is paucity of published information regarding pediatric neck masses in Tanzania and the study area in particular. This study determines the etiology, clinico-histopathological patterns and treatment outcome of pediatric neck masses and to identify predictors of outcome in our local setting.

**Methods:**

This was a prospective cross-sectional hospital based study done in children aged ten years and below with neck masses for a five months period. Statistical data analysis was done using SPSS version 17.0.

**Results:**

A total of 148 patients were studied. Their ages ranged from 2 months to 10 years (median 3 years). The male to female ratio was 2.5:1. Inflammatory lesions were the most frequent cause of neck masses accounting for 43.9% of cases. The median duration of illness was 2 years. Except for the neck mass, 72 (48.6%) of the children had clinically stable health condition on presentation. The posterior triangle was commonly involved in 118 (79.7%) patients. eight (5.4%) were HIV positive. The majority of patients (95.9%) were treated surgically. Postoperative complication rate was 30.4% and surgical site infection was the most frequent complication in 37.5% of cases. The median length of hospital stay was 10 days and was significantly longer in patients with malignant masses and those with surgical site infection (p <0.001). The overall mortality rate in this study was 8.1% and it was significantly associated with malignant masses, associated pre-existing illness, late presentation, HIV positivity, low CD 4 count, high ASA class and presence of surgical site infections (p <0.001). The outcome of patients on discharge was excellent as more than 90% of patients were successfully treated and discharged well.

**Conclusion:**

Pediatric neck masses are among the most common causes of paediatric surgical admissions and pose a diagnostic and therapeutic challenge in our setting. We advocate early surgical consultation and thorough and timely histopathological examination of neck masses in children.

## Background

Neck masses are a common complaint in children worldwide, and constitute a major indication for surgical consultation in many pediatric surgical centers [1, 2]. The occurrence of a mass in the neck during childhood creates anxiety for both parents and family physicians because of fear of probable underlying malignancy and frequently associated cosmetic problem [3]. This often results in early presentation [1, 2].

The etiology of neck masses varies from benign/congenital lesions which may be present at birth to acquired/neoplastic lesions which may manifest in late childhood [1–5]. The most common etiologies include congenital lesions and their complications, lymphadenopathy, vascular malformations, inflammatory and malignant lesions [6, 7]. The vast majority of neck masses in children are benign lesions [8, 9]. Despite this, special concern should be given for the possibility of a malignancy. Therefore, differentiation of benign lesions from malignant pediatric neck tumors and accurate definite diagnosis are essential for treatment planning as well as for prognosis for malignant tumors [10, 11].

Neck masses can present a diagnostic challenge in many resource-poor centers like in Tanzania where affected children often present late, and there may be a lack of sophisticated imaging facilities required for prompt and precise diagnosis [10, 12]. Consequently, although clinical assessment may result in straightforward diagnosis in many cases, histopathological examination of neck masses remains an important step in managing affected children even in centers with advanced diagnostic tools [12, 13]. Various advanced imaging modalities such as Ultrasound, Computed tomography (CT) and magnetic resonance imaging (MRI) are effective for noninvasive evaluation of a neck mass and its relationship to adjacent soft tissues and bony structures [10, 14, 15]. However, these diagnostic imaging are expensive and commonly not available in many centers in resource-limited setting.

The management of neck masses among paediatric population in our environment poses major challenges which need to be addressed. Late presentation with advanced lesion coupled with lack of sophisticated imaging facilities required for prompt and precise diagnosis are the hallmarks of the disease in our setting. The outcome of treatment of these patients in our setting has been poor because the majority of these patients present late to the hospital with advanced disease. This is partly due to paucity of local data regarding this condition, and lack of community awareness on the importance of early reporting to hospital for early diagnosis and treatment. An understanding of the patterns of these masses will improve diagnosis, preoperative decision making and their overall management. This study was intended to describe the pattern and treatment outcome of neck masses among paediatric patients treated at our centre and highlights predictors of outcome for these patients.

## Methods

### Study design and setting

This was a cross-sectional hospital based prospective study with a follow up component for a period of five months between November 2012 and April 2013. The study was conducted in the paediatric surgical wards and surgical outpatient clinic of Bugando Medical Centre. Bugando Medical Centre (BMC) is a referral, consultant and teaching hospital for the Catholic University of Health and Allied Sciences-Bugando (CUHAS-Bugando) and other paramedics and it is located in Mwanza city in the northwestern part of the United Republic of Tanzania. It is situated along the shore of Lake Victoria and has 1000 beds. BMC is one of the four largest referral hospitals in the country and serves as a referral centre for tertiary specialist care for a catchment population of approximately 13 million people from northwestern part of Tanzania.

### Study population

The study population included all paediatric patients aged 10 years and below presenting with neck masses in the paediatric surgical wards of BMC during the study period and whose parents or caretakers consented for the study. A neck mass was defined as any abnormal enlargement, swelling, or growth from the level of the base of skull to the clavicles. Patients who had no parents or caretakers to consent for the study and for HIV testing were excluded from the study. Convenience sampling of patients who met the inclusion criteria was performed until the sample size was reached.

Recruitment of patients to participate in the study was done at the surgical outpatient clinic and in the paediatric surgical wards after an informed written consent. Patients were screened for inclusion criteria and those who met the inclusion criteria were offered explanations about the study and requested to consent before being enrolled into the study. The diagnosis of paediatric neck masses was made based on clinical examination; radiological assessments as well as obtaining tissue for histopathological analysis. Fine needle aspiration biopsy (FNAB) was performed in any neck mass that is not an obvious abscess and persists following prescribed antibiotic therapies. Patients were also screened for HIV testing using Tanzania HIV Rapid Test Algorithm and CD 4+ count using FACS or FACSCALIBUR from BD Biosciences USA. A determination of CD 4 count was only performed in HIV positive patients. The research assistants and the principal investigator gathered relevant information regarding the history, physical examination, and outcome measures. All recruited patients were managed accordingly.

Patients scheduled to undergo emergency surgery were admitted through A&E department after thorough resuscitation, and patients scheduled for elective surgery were admitted a day before surgery through surgical outpatients.

Pre-operatively, all patients were assessed for fitness for surgery and anesthesia. Each patient was classified according to the physical status scale of the American Society of Anesthesiologists or ASA class, which assigns a risk level for surgery and anesthesia. Operative findings were noted and postoperative course and findings were noted. Postoperatively all patients were given a full dose of Parenteral narcotic analgesic such as Pethidine. Antibiotics such as gentamicin, cephalosporins ampiclox etc. were also prescribed in all patients. Daycare patients were discharged home on the day of operation after fully recovered from anesthesia. Inpatients were discharged two to seven days after surgery depending on the physiological status of the patients. All patients were reviewed on the seventh day after operation for stitches removal and examination of wound for evidence of wound sepsis. The Principal investigator and Research assistants ensured that the study patients were receiving the appropriate treatment and supportive care as prescribed by the surgeon. Patients were followed up until discharge or death. HIV positive patients were referred to the Care and Treatment Clinic (CTC) after counseling.

Data were collected using a pre-tested coded questionnaire. Data administered in the questionnaire include; socio-demographic characteristics (e.g. age, sex), anatomical distribution of the masses (anterior, posterior triangles), clinical presentation, radiological modalities and findings (Plain x-ray, ultrasound and CT scan of the neck), HIV status and type of treatment offered. The length of hospital stay, treatment complications and mortality as measures of outcome of these patients were recorded at the end of follow up period.

### Statistical data analysis

The statistical analysis was performed using statistical package for social sciences (SPSS) version 17.0 for Windows (SPSS, Chicago IL, U.S.A). The median and ranges were calculated for continuous variables whereas proportions and frequency tables were used to summarize categorical variables. Chi-square (*χ*2) test were used to test for the significance of association between the independent (predictor) and dependent (outcome) variables in the categorical variables. The level of significance was considered as P <0.05. Multivariate logistic regression analysis was used to determine predictor variables that predict the postoperative complications, hospital stay and mortality.

### Ethical consideration

Ethical approval to conduct the study was obtained from the CUHAS-Bugando/BMC joint institutional ethic review committee before the commencement of the study. An informed written consent was sought from the parents or guardians.

## Results

### Patient’s characteristics

During the study period, a total number of 152 patients were treated at our centre. Of these 4 patients were excluded from the study due to failure to meet the inclusion criteria. Thus, 148 patients were enrolled into the study. Of these, one hundred and six (71.6%) were males and 42 (28.4%) were females. The male to female ratio was 2.5: 1 with a male predominance in each age group (Table 1). The age of patients ranged from 2 months to 10 years with a median age of 5 years and Interquartile range of 2 to 7 years. The modal age group was 3–5 years accounting for 31.8% of cases. The majority of patients, 108 (73.0%) came from the rural areas located a considerable distance from the study area. Ten (6.4%) patients presented with history of pre-morbid illness which were congenital heart diseases in 3 (30.0%) patients, tuberculosis in 3 (30.0%) patients, rheumatic heart disease in 2 (20.0%) patients, nephrotic syndrome and sickle cell disease in 1(10.0%) patient each respectively.

**Table 1:** **Sex distribution according to age group**

Age group (years)	Sex		
	Male (N/%)	Female (N/%)	Total (N/%)
< 1	5 (3.4)	2 (1.4)	7 (4.8)
1-2	19 (12.8)	16 (10.8)	35 (23.6)
3-5	36 (24.3)	11 (7.4)	47 (31.8)
6-8	36 (24.3)	3 (2.1)	39 (26.4)
9-10	10 (6.8)	10 (6.8)	20 (13.6)
**Total**	**106 (71.6)**	**42 (28.4)**	**148 (100)**

### Etiological spectrum

In the present study, inflammatory lesions were the most frequent cause of neck masses accounting for 43.9% of cases. The median age of patients with congenital lesions was 3 years (range 1–9 years), while that of acquired lesions was 5 years (range 2–10 years). The difference in distribution of the congenital and acquired diseases by age was statistically significant (P <0.001) (Table 2). Cystic hygroma was the most common congenital causes (18.2%), whereas reactive lymph node hyperplasia was the most frequent cause of inflammatory neck masses (28.3%) (Table 3).

**Table 2:** **Etiological distribution according to age group**

	Causes					
Age group (in years)	Congenital (N/%)	Inflammatory (N/%)	Neoplastic (N/%)	Traumatic (N/%)	Other e.g. plunging ranula (N/%)	Total (N/%)
<1	7 (4.7)	0 (0)	0 (0)	0(0)	0 (0)	7 (4.7)
1-2	29 (19.6)	5 (3.4)	1 (0.7)	0 (0)	0 (0)	35 (23.6)
3-5	10 (6.8)	31 (20.9)	6 (4.1)	0 (0)	0 (0)	47 (31.8)
6-8	5 (3.4)	24 (16.2)	7 (4.7)	2 (1.4)	1 (0.7)	39 (26.4)
9-10	6 (4.1)	5 (3.4)	8 (5.4)	1 (0.7)	0 (0)	20 (13.5)
**Total**	**57 (38.5)**	**65 (43.9)**	**22 (12.9)**	**3 (2.1)**	**1(0.7)**	**148 (100)**

**Table 3:** **Distribution of neck masses according to etiology (N = 148)**

Etiology	N%
**Inflammatory lesions**	65 (43.9)
• React. cervical lymph node hyperplasia	42 (28.3)
• Neck abscess	13 (8.8)
• Tuberculous adenitis	8 (5.4)
• Chronic Sialoadenitis	2 (1.4)
**Congenital lesions**	57 (38.5)
• Cystic hygroma	27 (18.2)
• Thyroglossal cyst	22 (14.9)
• Dermoid Cyst	6 (4.1)
• Branchial cleft cyst	2 (1.4)
**Neoplastic lesions**	
**a. Benigntumors**	16(10.8)
• Hemangioma	6 (4.1)
• Fibroma	5 (3.4)
• Neurofibroma	4 (2.7)
• Lipoma	1 (0.7)
**b. Malignant tumors**	6 (4.1)
• Hodgkin’s lymphoma	3 (2.1)
• Non- Hodgkin’s lymphoma	2 (1.4)
• Soft tissue sarcoma	1 (0.7)
**Traumatic lesions**	3 (2.1)
**Other lesion eg** Plunging ranula	1 (0.7)
**Total**	148 (100)

### Clinical presentation

The duration of illness ranged from 1 day to 8 years with a median age of 2 years (IQR = 1 to 5 years) and the majority of patients 102 (68.9%) presented late between 1 and 3 years after the onset of the illness. Anorexia, weight loss, failure to thrive, anemia, and extensive scarifications of the mass by traditional healers were other presenting features in many children, especially those with malignant neck mass. Only 48 (32.0%) of the children who were at the early stages of the disease presented directly within the first week of noticing the neck mass. The reasons for late presentation are shown in Table 4.

**Table 4:** **Reasons for late presentation (N = 102)**

Reasons for late presentation	N%
Financial problem	98 (96.1)
Treated at peripheral hospitals	72 (70.6)
Lack of money for transport	70 (68.6)
Treated by traditional healers	23 (22.5)
Self medications at home	21 (20.6)
No reason given	15 (14.7)

All patients presented with neck swelling. The size of the mass ranged from 1 × 1 cm to 20 × 15 cm with 82 (55.4%) of masses having a size of 2 × 4 cm at presentation. Swelling was non-tender in 76 (51.4%) patients with cystic consistency in 70 (47.2%) patients, firm to hard in 62 (41.9%) and soft in 16 (10.8%) patients. The posterior triangle was commonly involved in 118 (79.7%) of the patients. Anterior triangle and both anterior and posterior triangles were involved in 22 (14.9%) and 8 (5.4%) patients respectively. The right side was frequently involved in 66 (44.6%) of the patients. This was followed by left side and bilateral involvement in 60 (40.5%) and 22 (14.9%) patients respectively. Associated symptoms and signs are shown in Table 5. Except for the neck mass, 72 (48.6%) of the children had clinically stable health on presentation, but 68(45.9%) were chronically ill and eight (5.4%) were severely compromised clinically in the terminal stages of the diseases. Forty-five (35.4%) patients had undergone some form of intervention in peripheral dispensaries and hospitals before referral, with dressing and inadequate surgical resection being the most common intervention.

**Table 5:** **Distribution of patients according to clinical presentations**

Clinical presentations	N%
**a. Symptoms**	
Neck swelling	148 (100)
Pain	68 (45.9)
Fever	45 (35.4)
Ulceration	40 (27.0)
Weight loss	35 (23.6)
Discharge	31 (20.9)
Failure to thrive	28 (18.)
Pressure symptoms	14 (9.5)
**b. Signs**	
Palpable neck mass	148 (100)
Tenderness	72 (48.6)
Wasting	70 (47.2)
Anemia	70 (47.2)
Ulceration	40 (27.0)
Discharge	31 (20.9)

### Investigations

Serological investigations for HIV infection revealed that out of 148 patients, eight (5.4%) were HIV positive. Of these, 2 (25.0%) patients were known cases on ant-retroviral therapy (ARV), and the remaining 6 (75.0%) patients were newly diagnosed patients. CD 4+ count, was available in 7 patients, which ranged from 142 to 420 cells/μl (median = 345 cells/μl + IQR of 178 to 420 cells/μl). A total number of three HIV infected patients (37.5%) had CD4+ count below 200 cells/μl, and the remaining 5 patients (62.5%) had CD4+ count of ≥200 cells/μ.

Plain neck radiographs was done in 126 (85.1%) patients and revealed abnormal findings such as tracheal/ mediastinal shift in 20 (15.9%) patients. Ultrasound was performed in 45 (30.4%) patients to determine the physical characteristics of the mass (i.e. whether solid or cystic). None of our patients had Computered Tomography (CT) scan, Doppler ultrasound or Magnetic Resonance Imaging (MRI) due to lack of these facilities at our centre.

Histopathological examinations (FNAB & open biopsy) were performed in all patients and revealed inflammatory lesions in 65 (43.9%) patients, congenital/developmental lesions in 57 (38.5%) patients and neoplastic lesions in 22(14.9%) patients (Table 3).

### Treatment

Out of 148 patients, 142 (95.9%) were treated surgically and the remaining 6 (4.1%) patients were treated conservatively with antibiotics such as cephalisporins, gentamicin and ampiclox etc. Of those who were treated surgically, surgical excision of the mass was the most common surgical procedure in 104 (73.2%) patients followed by Sistrunk's operation in22 (15.5) patients and incision and drainage of neck masses in 13 (9.2) patients. Other surgical procedures (such as incisional biopsy) was performed in 8 (5.6) patients. In addition to surgery, eight (5.6%) patients of histologically confirmed tuberculosis were treated with anti-tuberculous drugs. Chemotherapy was prescribed in five (3.4%) patients who had lymphoma. All patients who were scheduled for operation were assessed pre-operatively using the American Society of Anesthetists (ASA) pre-operative grading. The majority of patients had ASA class II accounting for 57.0% of cases.

Children with cystic hygroma who presented with respiratory compromise due to superimposed infection and huge confluent mass had initial incision and drainage/antibiotics and repeated aspiration respectively, before definitive surgical excision was undertaken. Sistrunk's operation gave excellent outcome in those with thyroglossal duct cyst, while additional combination antituberculous chemotherapy was used in those with tuberculous lymphadenopathy.

### Treatment outcome

A total of 48 postoperative complications were recorded in 45 patients giving a complication rate of 30.4%. Surgical site infection was the most common postoperative complications accounting for 37.5% of cases (Table 6).

**Table 6:** **Distribution of patients according to postoperative complications (N = 48)**

Postoperative complications	N%
Surgical site infection	18 (37.5)
Bleeding	10 (20.8)
Wound dehiscence	5 (10.4)
Recurrence	9 (18.7)
Septicaemia	3 (6.3)
Pneumonia	2 (4.2)
Urinary tract infection	1 (2.1)

The overall length of hospital stay (LOS) ranged from 1 day to 56 days with the median of 10 days + IQR = 6 to 14 days. The LOS for non-survivors ranged from 1 day and 12 days with a median of 5 days + IQR of 2 to 7 days. Patients with complications (*p* = 0.023) and those who had malignant neck swelling (*p* = 0.001) stayed longer in the hospital.

Out of 148 patients, 12 patients died leading to mortality rate of 8.1%. According to multivariate logistic regression analysis, malignancy cause (OR =1.3, 95% CI =1.1-4.6), p = 0.001), pre-existing illness (OR =3.4, 95% CI =2.2- 9.0), p = 0.014), late presentation (OR =2.3, 95% CI =1.6 -6.2, p =0.011), HIV positivity (OR =6.3, 95% CI =2.2 -12.9, p = 0.003), low CD 4+ count (OR =4.2, 95% CI = 0.6, 95% CI = 0.2- 0.9, p = 0/000), high ASA class (OR =8.1, 95% CI (5.6-12.9), p = 0.014) and surgical site infection (OR = 6.7, 95% CI = 2.5- 8.9) p = 0.022) were the main predictor of mortality.

Out of 136 survivors, 130 (95.6%) were treated successfully and discharged well and the remaining six (4.4%) patients were discharged themselves against medical advice. No patient in this study was discharged with permanent disability. We could not establish the reason for the discharge against medical advice.

## Discussion

Pediatric neck masses are among the common surgical condition presenting to the pediatric surgical wards and clinics worldwide, and contribute significantly to morbidity and occasionally mortality [1]. In this study, the majority of patients were aged between 3–5 years which is comparable to other studies which reported similar age group [3, 6]. We could not establish the reason for the high incidence of pediatric neck masses in this age group.

In agreement with other studies [3, 4, 6, 15], males were more affected than females with a male to female ratio of 2.5: 1. We could not find the reason for this gender predilection.

The majority of our patients in this study came from the rural areas located a considerable distance from the study area. Similar observation was reported by others [3, 6]. This observation has an implication on accessibility to health care facilities and awareness of the disease.

In the present study, inflammatory lesions were the most frequent cause of neck masses among pediatric population which is in agreement with other studies [1, 15], but other authors reported that congenital lesions was the most common causes of pediatric neck masses [3, 6–8]. The reason for this etiological pattern is not clear. Inflammatory lesions are the most common neck masses and this is supported by our own study. Inflammatory lesions take first place among neck masses in developing countries while congenital and neoplastic masses are most common in developed countries [2, 3].

As reported by others [4, 5, 10], patients with congenital neck masses in the present study were found to be younger than those with acquired neck masses. This observation can be explained by the fact that congenital neck masses usually present during birth whereas acquired neck masses present later in life.

The commonest congenital neck masses in this study were cystic hygroma which accounted for 18.2% and thyroglossal duct cyst which accounted for 14.9% of cases. This finding corresponded with the reports of earlier authors [3, 6, 9] but differ with other study which reported thyroglossal duct cyst as the commonest congenital neck masses [7, 11]. Like other studies [3, 6], reactive lymph node hyperplasia was the most frequent cause of inflammatory neck masses in this study.

In the present study, more than two third of patients presented late to the tertiary health facility. Studies done in other developing countries showed that financial constraints and treatment at peripheral hospitals were the most common reasons for the late presentation [3, 6, 16] which is also similar in this study. Moreover, in this study, late presentation resulted in 45.9% cases arriving in chronically ill condition, with 5.4% in terminal stages of the diseases which corresponded with earlier observations in this African sub Saharan region [3, 6]. Despite obvious location of the masses, anorexia, weight loss, failure to thrive, and anemia had supervened in many children and these were the indications for seeking surgical consultation in many cases, as also reported by others [6, 15]. The use of scarification and application of native concoction remain common modality of treatment of many ailments by African traditional healers. Prior employment of this modality of treatment was obvious in these children as extensive scarification marks were present on arrival in many children, especially those with malignant neck masses [6].

The posterior triangle was commonly involved in more than three quarter of patients. Similar anatomical distribution was also reported by others [11–15]. High incidence of lesions in the posterior triangle in the present study may be explained by the fact that the majority of lesions were inflammatory in origins which are usually located in the posterior triangle.

The prevalence of HIV infection among patients with pediatric neck masses in the present study was 5.4%, a figure which is low as compared to the figure of 6.5% in general population. Low HIV seroprevalence in our study can be explained by the fact that the study included only pediatric population who usually not at risk of getting HIV infection compared to adults. The HIV prevalence in children aged less than 10 years of age is most likely caused by vertical transmission or by blood transfusion.

Neck masses in children can be evaluated by various imaging modalities. Ultrasound is usually the initial imaging modality used for locating and identifying a neck mass and for determining solid versus cystic components. Computed tomography (CT) and magnetic resonance imaging (MRI) are effective imaging techniques for noninvasive evaluation of a neck mass and its relationship to adjacent soft tissues and bony structures. MRI has distinct advantages over CT, since it uses no ionizing radiation, has multiplanar capability, and has much better intrinsic soft-tissue contrast. MRI takes more time to image, this which can be difficult in pediatric patients. The imaging modalities selected should complement the clinical evaluation of the neck mass in a patient. In the present study plain neck x-ray and ultrasound was the only imaging modalities of choice. None of our patients had Computer Tomography (CT) scan, Doppler ultrasound or Magnetic Resonance Imaging (MRI) due to lack of these facilities at our centre.

In agreement with other studies, surgical excision of the neck mass was the most common type of surgical procedure performed in the present study [2, 3, 5, 16, 17]. Similarly, Sistrunk's operation and additional combination antituberculous chemotherapy gave excellent outcome in those with thyroglossal duct cyst and tuberculous lymphadenopathy, respectively, as reported in earlier studies [3, 6].

The place of physiotherapy in managing both malignant and nonmalignant neck masses, especially following surgical excision, cannot be overemphasized. In present review, all the residual postoperative neck deformity responded to physiotherapy, with significant improvement during follow-up. This is similar to earlier reports in which physiotherapy and reassurance of parents resulted in favorable outcome of postoperative neck deformity [18–21].

The presence of complications has an impact on the final outcome of patients presenting with neck masses. In keeping with other studies [3, 4, 6, 18], surgical site infection was the most common postoperative complications in the present study. High rate of surgical site infection in the present study may be attributed to HIV seropositivity and low CD 4 count.

The overall median duration of hospital stay in the present study was 10 days which is higher than that reported by other authors [22, 23]. Patients with complications and those who had malignant neck swelling stayed longer in the hospital. However, due to the poor socio-economic conditions in Tanzania, the duration of inpatient stay for our patients may be longer than expected.

The overall mortality rate in this study was 8.1% and it was significantly associated with malignant cases, associated pre-existing illness, late presentation, HIV positivity, low CD 4 count, high ASA class and presence of surgical site infections. Addressing these factors responsible for high mortality in our patients is mandatory to be able to reduce mortality associated with this disease.

## Conclusion

Pediatric neck masses remain the most common causes of pediatric surgical admissions to Bugando Medical Centre. Late presentation coupled with lack of sophisticated imaging facilities required for prompt and precise diagnosis and delayed acquisition of histopathological results are the hallmarks of the disease in our setting. We recommend the need for public enlightenment campaign on the importance of early referral for surgical consultation in this sub-region. Efforts should also be made to subject all neck masses to histopathological examination as some of the healthy looking children were found to have malignant lesions.
